# Developing a mHealth intervention to promote uptake of HIV testing among African communities in the conditions: a qualitative study

**DOI:** 10.1186/s12889-016-3278-4

**Published:** 2016-07-28

**Authors:** C. Evans, K. Turner, L. S. Suggs, A. Occa, A. Juma, H. Blake

**Affiliations:** 1 School of Health Sciences, University of Nottingham, A Floor, South Block Link, Queen’s Medical Centre, Nottingham, NG7 2HA United Kingdom; 2 BeCHANGE Research Group, Institute for Public Communication, Faculty of Communication Sciences, Università della Svizzera italiana, Lugano, Switzerland; 3 School of Communication, University of Miami, Miami, USA; 4 African Institute for Social Development, Nottingham, United Kingdom

**Keywords:** HIV testing, mHealth, Text messaging, African, Community-based participatory research, Social marketing

## Abstract

**Background:**

HIV-related mHealth interventions have demonstrable efficacy in supporting treatment adherence, although the evidence base for promoting HIV testing is inconclusive. Progress is constrained by a limited understanding of processes used to develop interventions and weak theoretical underpinnings. This paper describes a research project that informed the development of a theory-based mHealth intervention to promote HIV testing amongst city-dwelling African communities in the conditions.

**Methods:**

A community-based participatory social marketing design was adopted. Six focus groups (48 participants in total) were undertaken and analysed using a thematic framework approach, guided by constructs from the Health Belief Model. Key themes were incorporated into a set of text messages, which were pre-tested and refined.

**Results:**

The focus groups identified a relatively low perception of HIV risk, especially amongst men, and a range of social and structural barriers to HIV testing. In terms of self-efficacy around HIV testing, respondents highlighted a need for communities and professionals to work together to build a context of trust through co-location in, and co-involvement of, local communities which would in turn enhance confidence in, and support for, HIV testing activities of health professionals. Findings suggested that messages should: avoid an exclusive focus on HIV, be tailored and personalised, come from a trusted source, allay fears and focus on support and health benefits.

**Conclusions:**

HIV remains a stigmatized and de-prioritized issue within African migrant communities in the UK, posing barriers to HIV testing initiatives. A community-based participatory social marketing design can be successfully used to develop a culturally appropriate text messaging HIV intervention. Key challenges involved turning community research recommendations into brief text messages of only 160 characters. The intervention needs to be evaluated in a randomized control trial. Future research should explore the application of the processes and methodologies described in this paper within other communities.

**Electronic supplementary material:**

The online version of this article (doi:10.1186/s12889-016-3278-4) contains supplementary material, which is available to authorized users.

## Background

Promoting uptake of HIV testing is recognized as a key priority in global HIV programming. In many countries, HIV prevention efforts are hindered by high levels of undiagnosed individuals and high levels of individuals diagnosed ‘late’ (defined as having a CD4 count of less than 350 cells per mm^3^ within 3 months of diagnosis and associated with significantly heightened levels of HIV-related morbidity and mortality) [1, 2]. The WHO and UNAIDS have endorsed a new global goal for 2020, specifically for 90 % of those with HIV to be diagnosed, 90 % of those diagnosed to receive ART and 90 % of those on ART to have a suppressed viral load. In order to achieve the first 90 % target around diagnosis, they have called for an expansion of existing HIV testing strategies, but also for the development and evaluation of new approaches, particularly community based approaches [3].

In the UK, as in much of Europe, African migrant communities are a priority population for HIV prevention efforts [4, 5]. Latest statistics estimate that 4.1 % of heterosexual black African men and 7.1 % of heterosexual black African women in the UK are HIV positive [5]. Of these, 38 % of men and 31 % of women are unaware of their diagnosis, and this figure is estimated to be even higher outside London (50 % of men and 41 % of women) [5]. These figures account for continuing high rates of late diagnosis within African communities. For example, in 2012 in the UK, 61 % of African women and 66 % of African men with HIV were diagnosed late [6]. A recent survey has estimated the annual HIV testing rate amongst African migrants in the UK to be 36.8 % [7]; however this number rises to 97 % amongst pregnant women attending ante-natal clinics [5].

The UK Health Protection Agency (HPA) recently conducted a series of pilot projects to evaluate the feasibility of HIV testing in ‘non-traditional’ settings [8]. These showed that the highest positivity rates were reported in the community based projects, leading the HPA to conclude that “*community based pilots*, *targeting most at risk populations*, *were shown to be highly acceptable and resulted in high numbers of individuals being newly diagnosed with HIV infection and transferred into care. Community HIV testing services need to be appropriately targeted and established with strong community representation*” [8:1]. They suggested that more evidence was required regarding the most effective combination of strategies that community organizations should adopt to encourage testing.

One potential strategy for enhancing the effectiveness of community based HIV testing programmes may lie in the use of new technologies, such as mobile phones [9]. In practical terms, mHealth interventions can be inexpensive and wide-reaching in application, and have demonstrated potential for reaching large samples and accessing hard-to-reach groups [10–15]. Much of the existing evidence on mHealth and HIV has examined the role of short message service (SMS) interventions in supporting HIV care once a diagnosis has been made (rather than to promote testing), for example, providing appointment reminders [16, 17], enhancing treatment adherence [18–22] and promoting retention in care [23–26]. Overall, there are strong indications that SMS/text messaging interventions can be effective [27], but the evidence remains somewhat mixed, with some studies reporting significant increases in adherence [18, 20, 28] and others reporting no benefit [29–31]. There is still much to be learned about the structure, content, tone and frequency of messages and the mechanisms by which they influence outcomes [19, 32, 33].

The evidence base for mHealth HIV prevention research remains limited but is yielding exciting results [11, 15, 34–36]. Several pilot and demonstration projects have utilized text messaging interventions for HIV-related health promotion, predominantly combining information-giving with promotion of HIV testing [37–39]. Outcomes relating to knowledge, changes in risk assessment and testing behavior have usually been measured through proxy indicators (such as calls to help-lines or changes in uptake of HIV testing in local clinics) [40]. However, two recent small scale randomized controlled trials in Kenya [35] and South Africa [36] have suggested that SMS interventions may have a direct impact on encouraging HIV testing behaviors.

To date, most research on SMS-based HIV prevention has been conducted in low and middle income countries [11, 15], with only a few studies reported from higher income contexts, primarily the USA [22, 39, 41]. Despite promising initiatives in other settings and with other populations, the efficacy of this approach amongst African migrant communities living in high-income countries has yet to be demonstrated. Furthermore, there is limited evidence documenting the message and intervention development process underpinning HIV-related mHealth interventions [42–46]. Indeed, lack of process-related evidence has been a common criticism of m-health interventions in general, threatening intervention transferability and hindering the development of more theoretically informed understandings of implementation processes.

Evidence on effective community-based programs suggests that the social marketing approach has been most effective in achieving positive outcomes, as well as recruitment and community engagement [47, 48]. Community-based social marketing requires that before the efficacy of an intervention can be established, development and feasibility work is required to ensure that the intervention meets the needs and expectations of users, and to ensure that the procedures associated with the intervention delivery and research processes (e.g. health or behavioral outcomes, methods of data collection) are appropriate [49, 50].

This paper describes the research and development processes used to identify the key HIV-related issues reported by African migrants in the UK and to inform the development of an SMS intervention entitled ‘Health4U’. First, it reports the research that was collaboratively undertaken to explore views within Nottingham’s African communities on HIV and HIV testing, on the proposed mHealth intervention and on appropriate message content, structure, language and frequency. Second, it demonstrates how these insights were used to collaboratively design and pilot a culturally appropriate, locally relevant and theory-informed SMS intervention. Quantitative outcomes are reported elsewhere [51].

## Methods

The development of the intervention utilized a number of innovative processes, specifically the co-creation of the intervention and a theory-informed approach, implemented through a community-based participatory social marketing process.

### Results

The study was conducted in the city of Nottingham in the East Midlands region of the UK with a population of approximately 314,300. Nottingham is considered to have a ‘high’ HIV prevalence (2.78 per 1,000 population), higher than both the regional (East Midlands) and national (England) averages. Between 2010-2012, 65.8 % of new HIV diagnoses in the city were made late, much higher than the England average of 48.3 % [52]. HIV testing is available free of charge through public sector (NHS) sexual health clinics in hospital, primary care and community settings. Some voluntary sector agencies have also been commissioned to undertake community-based work with specific target groups. The purchase of home testing kits has recently been made legal in the UK – this approach was being piloted nationally at the time of the study.

A recent report estimated an African migrant population in Nottingham of approximately 5,000, representing 31 different countries [53]. This is a highly mobile population so exact population figures are difficult to calculate. African communities in Nottingham are represented by over 13 nationality-based community groups, faith-based groups and some pan-African community-based organizations. One of the latter is the African Institute for Social Development (AISD), with considerable experience in HIV prevention work.

### Research design

The study adopted a social marketing approach using community based participatory research (CBPR) to design the messages and intervention structure [54]. Unertl et al [45:1] defined CBPR as “*a collaborative*, *action orientated research approach that involves the development of long term*, *equitable research partnerships between academic researchers*, community based organisations (CBOs) and community members”. Community-based Social Marketing is “*based upon research in the social sciences that demonstrates that behavior change is most effectively achieved through initiatives delivered at the community level which focus on removing barriers to an activity while simultaneously enhancing the activities benefits*” [50:543]. The strength of this type of research is that all the partners involved in the process contribute to the project with their expertise, allowing the team to meet the specific needs of the community, in a culturally appropriate way [45, 55, 56]. CBPR, when authentically conducted, has been shown to yield significant benefits both for the research as well as the community to whom it is directed [45, 56]. These benefits have been defined as: more relevant research, wider impact, better fit between interventions and target beneficiaries, more effective recruitment and retention of diverse populations, possibility to access difficult-to-reach groups of people, improved internal validity, more rapid translation of research into action and development of people [45].

Consistent with community-based social marketing, our study sought to apply CBPR principles at every stage. From the outset, the project was undertaken as a partnership between the AISD and a university-based research team. AISD recruited a community research team to work on the project on a pro rata basis. The community team comprised 12 community researchers who were given in-depth training on research methods, mHealth initiatives and design principles, recruitment strategies, research ethics and HIV [56]. The overall project team had expertise in design and delivery of SMS-based interventions [57–59], and collectively included expertise from health psychology, health communication, social marketing, community organizing and lived experience of HIV.

**Fig. 1:**
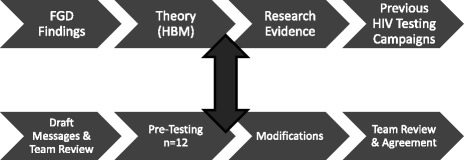
Message development process

The research involved focus groups (FGs) with African community members, moderated by pairs of community and university researchers. The goal of the FGs was to solicit knowledge, attitudes and beliefs about HIV, HIV testing, and text messaging for the purpose of HIV testing promotion. Subsequent intervention design and message development were informed by the FG findings and undertaken collaboratively. Messages were then pre-tested with individuals from the targeted African communities. A summary of the overall research and intervention design process is provided in Fig. 1.

### Theoretical framework

A common criticism of the existing literature reporting SMS-related health interventions has been that they lack a theoretical framework [60]. To address this weakness, the study was informed by the ‘Health Belief Model’ (HBM) [61–63] which has been used to explain a variety of long- and short-term health behaviors, including sexual risk behaviors and the transmission of HIV/AIDS [43]. The HBM was developed to explain why individuals at risk of contracting tuberculosis (TB) did not participate in free TB screenings [64], and continues to be used to explain why people get tested for a variety of diseases. It has commonly been used to explain HIV testing and HIV prevention behaviors [35, 43, 65, 66]. The HBM was used in this study to inform message design and to test outcomes. Specifically, the intervention was cognizant that individual’s perceived susceptibility to contracting HIV may be low, the perceived benefit of testing may be low, and the perceived severity of being tested as a migrant may be high [67, 68]. Furthermore, cues to action could be used to change these perceptions and to promote HIV testing. These elements were considered in the design of messages.

### Research and message pre-testing: sampling and recruitment

Six FGs were held, and utilized purposive sampling to represent the social and religious characteristics within Nottingham’s African communities, as follows: Muslim men (MM), Muslim women (MW), Christian group (CG), Community leader group (CL), younger person’s group (under 30 years) (YP), mixed group (not an active Christian or Muslim, not a community leader, and over 30 years) (MG). The sampling strategy aimed to capture the views of the target population for the proposed intervention (i.e. the general African migrant community), rather than differentiating this population further, for example, on parameters related to previous HIV testing history. It was recognized that perceptions of HIV testing and personal risk could be shaped by previous testing outcomes and experiences. However it was felt that in order to facilitate open discussion about HIV in the groups, they needed to be relatively homogeneous and based around features of shared identity (such as gender, religion or age) rather than experience of HIV tests [69]. We felt that the diversity within the focus groups would enable the findings to reflect a wide range of views and experiences in this area.

Recruitment for the FGs was undertaken by the trained community researchers via voluntary sector groups, local community venues (e.g. churches/mosques), nationality based community groups and via external contacts (e.g. library, hairdressers, advertising in local media). Individuals were provided with a flyer about the project and were invited to a FG at a pre-set time and date. Participants received a £20 (US$31) voucher for their time. Food and childcare were provided.

Recruitment for the pre–testing of messages followed the same process. Twelve individuals took part in the message pre-test (two from each group above).

### Data collection

The FGs were co-facilitated by a community and a university researcher. The discussions were recorded and transcribed. Most FGs lasted between 1-2 h. The ‘Muslim Women’s’ FG was undertaken in Arabic; the rest were conducted in English. The FGs were structured using a pre-set topic guide (see Additional file 1). Participants were also shown a video clip from an existing text-messaging intervention (Text4baby [70]) to provide them with a more concrete idea of what kind of intervention was being proposed.

The message pre-testing process followed an established elicitation interviewing method [57, 59]. During this process, individuals were asked to read each of the text messages and to answer a set of questions for each individual message and for the group of messages as a whole (see Additional file 1).

### Data analysis, message development and pre-testing

FG data were analyzed thematically using a framework approach [71, 72]. Transcripts were coded in NVivo, and a set of initial descriptive themes was developed. Where possible, these were mapped against constructs from the HBM and then further refined [43]. Analysis was undertaken by the university team and the emerging interpretations were discussed and agreed upon with the community research team [73].

Message development was based on: (i) an in-depth review of the FG findings (categorized thematically within the main HBM constructs), (ii) a review of messages used in previous and existing HIV testing interventions, and (iii) lessons drawn from the existing evidence base [36]. Messages were developed as a team, using an iterative process of writing, review, pre-testing and further modification until a final version was agreed upon. The final set of messages was also reviewed by the clinical lead for HIV in the city and a large HIV-related voluntary agency. A similar team-based approach was used to translate the messages into Arabic and French, using a combination of conceptual translation [74, 75] and back translation approaches [76]. Initial and back translations were carried out by independent translators. These were then reviewed together with members of the team (community researchers for whom French and Arabic were first languages, and academic researchers) who knew what ‘meaning’ the messages were attempting to convey, and who provided final cross-checking and suggestions for relevant modifications [77].

## Results

The main results of the research are presented thematically in accordance with the Health Belief Model (Table 1). An additional category ‘*Views on Proposed SMS Intervention*’ is also presented. Illustrative quotes illuminate key concepts. Identifiers from the different FGs are used in brackets to show the origin of each quote.

**Table 1:** Thematic analysis framework

Health belief model construct	Themes
Perceived Susceptibility to HIV	• Community-defined risk
	• Unfair targeting
	• Invulnerability
Perceived Severity of HIV	• Awareness of HIV
	• Fear of consequences
	• Social stigma and taboo
Perceived Benefits for HIV Testing	• Staying healthy
	• Receiving support
	• Protecting others
Perceived Barriers for HIV Testing	• Lack of knowledge about testing issues
	• Accessibility of services
	• Culture of health seeking
	• Complex lives
Self-efficacy	• Trust in health providers
	• Trust and support of communities
Cues to Action	• Visibility and awareness raising
	• Personalisation and targeting
Views on the Proposed SMS Intervention	• Perceived benefits and concerns
	• Message content
	• Intervention structure

### Participant characteristics

The 6 FGs included a total of 48 participants. There were 19 men and 29 women, representing 19 different African countries, ranging in age from 18-45 years. Participant characteristics are provided in Table 2.

**Table 2:** Demographic characteristics of focus group participants

Group	Median age	Gender	Countries of origin
Mixed Group	37.5	6 Males, 3 Females	Algeria, Cameroon, Eritrea, Gambia, Ghana, Morocco, Nigeria, Rwanda, Swaziland Uganda
Muslim Women	34	10 Females	Algeria, Egypt, Eritrea, Morocco, Sudan, UK
Community Leaders	40.2	6 Males	Egypt, Ghana, Ivory Coast, Jamaica, Morocco
Muslim Men	38.2	5 Males	Gambia, Jamaica, Somalia, Sudan
Young Persons Group	20.2	2 Males, 4 Females	Congo, “Africa”
Christian	38.3	12 Females	Eritrea, Kenya, Malawi, Nigeria, Uganda, Zimbabwe

### Perceived susceptibility to HIV

Perceived susceptibility was discussed in relation to individual notions of risk, and risk associated with ‘place’ and community.

Groups readily recognized that individual risk was associated with individual behavior and primarily linked to sexual transmission. However, a common theme was that even though individuals may be aware of HIV, a human tendency towards perceived invulnerability hindered the translation from an abstract notion of risk to a personal sense of risk, especially in situations where individuals may not have had any direct experience of HIV:

“Y*ou may know that there are some diseases out there and you may hear about them from the TV* - *but you never think that YOU might catch one of them*…..*I don*’*t know anyone suffering from AIDS*” [*MW*, *R3*]

As noted above, perceptions of risk were shaped by previous personal experiences of HIV, which for many, had been in Africa, and there was a sense in which risk was associated with life in Africa rather than the UK. Participants across the groups expressed a lower awareness and some denial that HIV was a significant health problem for the African community in the UK:

“It is scaring people in Africa, but here it is not common. I have never seen a HIV patient dying here. I don’t know anyone suffering from AIDS - I don’t think it’s a problem is it?” [CL, R3]

In addition to place-based notions of risk, perceived susceptibility was also linked to a sense of community and religious affiliation and the perceived risk of HIV within these particular groupings. For example, participants in the Muslim FGs expressed a common view that their religion and culture were protective against HIV due to low perceived prevalence of HIV and also low perceived prevalence of related risk behaviors within their communities:

“In England, Nottingham, we don’t have as much of a problem with AIDS because…it is not designed for here, it is designed for out there, so it leaves here and go there and this is why a lot of us as Muslim it doesn’t all really affect us.” [MM, R5]

Similarly, for Christian participants, a key theme was the difficulty of addressing risk in a community context where sexual activity outside marriage was frowned upon:

“*It is difficult for pastors*/*priests to talk about AIDS when they as Christians obviously one of the teachings of the church is that you have to be faithful you know*, *so it could be really tricky when it comes to the church*, *sensitizing people*, *telling people about AIDS*” (*YP*, *R1*)

A related theme within all the groups was suspicion about the appropriateness of, and motives for, community-focused targeting. For example, participants questioned the relative risk of Africans having HIV compared to other nationalities, which revealed a sense of uncertainty around ‘official’ prevalence rates. There was a strong view that *any* nationality was at risk of having HIV and that Africans alone should not be singled out:

“I would disagree with anybody who says ”we’re targeting Africans because HIV is an African thing”....... what about people from outside? They can get HIV and come to us?..........…I’m not sure if statistics can “prove” that Africans here suffer more from HIV compared to other continents.” [CL, R6]

A sense of unfair targeting was linked to a wider theme (discussed in more depth below) of a lack of trust in mainstream healthcare and, therefore, a suspicion of official health advice. This suspicion was illustrated in some groups in terms of how health promotion messages around HIV were interpreted:

“To be honest with you I don’t believe [the statistics on prevalence]. We are from Africa and we know the culture there, similar - every country similar - there is no big difference”. [MM R1]

Together, these themes highlight that perceived susceptibility to HIV was shaped by individual experiences and social identities constructed through association with community, religion and place.

### Perceived severity of HIV

This category illustrates how perceived severity was influenced by fear of the consequences of HIV – expressed in terms of health consequences and social consequences.

All groups shared a common perception that HIV was a serious disease which could lead to sickness or death.

“Most people think it is still a deadly illness. They are scared to get tested. There is that fear of knowing that I actually have it and you know that there is no cure, so you have that - ah I don’t want to know that I am going to die” (MG, R6)

This fear was attributed to a lack of up to date or sufficiently detailed information about contemporary developments in HIV care and prognosis, and also to past experiences of witnessing HIV disease in Africa.

Fear was also strongly associated with the perceived social consequences of HIV due to its enduring social stigma. Participants noted that stigma created fears around potential loss of social support and made it difficult for communities to openly engage with HIV-related issues:

“People might think ”if I get to find out that I have this illness the whole family will like leave me”, so I think it is, you know, the fear of the unknown really… This [HIV] is very sensitive, and I think sometimes people within their community are very embarrassed to talk about it or ask questions about it.” [YP, R1]

Hence, perceived severity was associated with deep-seated fears of the consequences of HIV, which creates challenges for HIV testing efforts and highlights a need for up to date knowledge and community action.

### Perceived benefits of HIV testing

Across FGs, the strongest identified benefit to HIV testing was health-related, and stressed the ability to access treatment and stay healthy.

“If we made them aware, they might be able to go and check themselves to know that even with HIV, they can live longer by taking the correct tablets and through a normal diet and stuff like that so that is my view on that one. People think that it is still a deadly illness. Maybe it is not curable - but now it is manageable, so people need some information about it” [CL, R1]

Another theme highlighted social benefits. For example, the groups noted that linking HIV testing to social support would provide a positive and reassuring message:

“It would help to tell people what support or treatment they will get if they’re diagnosed with disease. Most people will tend to be scared, they think “oh if I have it, no one will want to come near me” sort of thing, so knowing what support they will get after, would be good.” [YP, R6]

Social benefits of HIV testing were also constructed in terms of potentially protecting others within the community or family. Participants therefore suggested that messages could be framed in terms of a positive appeal to an individual’s sense of responsibility:

“You can write “think about your kids” or “think about your future” in the message.” [MW, R1]

The themes in this category were closely related to the perceived severity category, which highlights how reported fears of the health and social consequences of HIV could be potentially re-framed.

### Perceived barriers to HIV testing

The key themes within this category were the most extensively discussed within the FGs. They were related to a lack of knowledge around HIV, to being a migrant in a new country and healthcare system, and to different cultural norms.

Participants indicated that a lack of knowledge in 2 areas discouraged people from getting an HIV test. This included (i) a lack of knowledge about HIV itself (in relation to its symptomatology and the fact that testing was important even in the absence of any symptoms), and (ii) in relation to the HIV testing process and services in the UK. For example:

“You know people think their health is fine; it is a kind of misconception… I think people are generally not aware of where these centers are; they do not know that the test is free so they worry about the cost.” [CL, R6]

Although HIV testing is free for any category of migrant in the UK, it was noted that not all migrants would be aware of this. In addition, there was a common assumption amongst the groups that HIV testing was primarily undertaken by the General Practitioner (GP) who was perceived as hard to access (due to waiting times or inflexible working hours), indicating lack of awareness of the many alternative options for HIV testing that are available:

“I think what stops me sometimes from going for a medical check-up, it is either work or other commitments and sometimes it takes too long to see a doctor…….sometimes 2 weeks……. it is a discouraging factor.” [YP, R1]

Another barrier to testing was fear around confidentiality within the testing process. This was attributed to HIV-related stigma and to a lack of familiarity with the structure and ethos of UK healthcare provision. Participants stressed that messages would need to emphasize how confidentiality would be maintained:

“It is all about making people understand that the test is going to be confidential - some people are actually scared of their confidentiality - they thought it might go. If confidentiality can be widely publicized you know within our communities… I think that would be a big, big, big headway” (CL, R5)

Participants related the above factors to the wider experiences of life as a migrant in the UK. They identified a range of complex and intersecting structural and emotional factors (such as lack of time, fears about immigration status, financial pressures, housing difficulties) that presented day to day challenges for individuals. When combined with mistrust in, and lack of familiarity of, the healthcare system, it was reported that these factors could all act together as barriers to seeking healthcare/HIV testing:

“They are worried about other issues you know immigration, work, family back home. You know they are worried about these things and maybe health is not a first priority for them.” [CL, R5]

Groups also identified that cultural and religious norms around health-seeking could act as a barrier to testing, noting that there was little tradition within African communities of seeking medical care unless someone was very ill. Participants explained that preventive health seeking when asymptomatic was not common practice:

“I don’t have that tendency to go to the hospital when I am not… you know when I am well, I don’t think I need to go to the hospital so…”[CG, R1]

Participants suggested that the expense and unavailability of health care services in their countries of origin may have reinforced these attitudes:

“It is expensive to go to GPs in our countries; we might experience pain in early stage but we don’t have money. We will only go when more complications occur.” [MW, R7]

In addition, some groups suggested that there was a cultural valorization of being strong and resilient (particularly for men):

“African men like to be seen as strong and tough - going to the hospital means you are weak - it’s a mentality problem.” [MM, R3]

Others noted that religious individuals might delay seeking formal care, preferring instead to rely on their faith practices for protection or healing:

“Even if you are dying, you see yourself with added strength and especially with Christian mentality, you don’t want to say you’re sick even if you’re feeling sick.” [CG, R4]

### Self-efficacy

A key factor influencing self-efficacy around testing concerned a lack of trust. This was expressed as lack of trust in healthcare providers and a lack of trust in being able to access a supportive community context.

Lack of trust in the healthcare system was, as noted above, linked to the experience of being a migrant. All groups demonstrated significant ambivalence towards health care staff. Concerns focused around perceived deficiencies in the quality of the service (e.g. complaints of long waiting times or consultations that were too short), or the competence of the provider (e.g. not prescribing the desired medications or the lack of physical examination of patients). Together, these led to an underlying sense of suspicion about the quality of care being provided:

“I think people are suspicious of the system, you know that even when you’re supposed to get screening and go for certain tests you don’t want to go because you don’t know what to expect, you know you’re not confident enough to trust anyone because they are not of your cultural group and it is frightening.” [CL, R2]

The suspicion of receiving poor quality care was also linked to a feeling of discrimination related to being African and/or black:

"People are scared [to get screened] because they know… for a long time black people are the first group to be likely to be lied to, be misdiagnosed, right? And mis-prescribed, wrong prescription so that is why they are very… they hold back when it is time to do those things." [CL, R4]

Linked to the above, perceptions of poor quality care also included a sense of being stereotyped and of not being treated like an individual. For example, a number of participants suggested that health care services pigeon-holed African people, and offered them HIV or other screening tests in order to meet targets or due to the assumption that they had HIV:

They put us in a pigeon hole as black women, and because we are in this kind if pigeon-hole, they want to get us through as black women – as many of us as possible - so they are seen to have done their work. But at the same time it is not about testing, it is not about me, it is about targets” [CG, R2]

The development of good relationships within healthcare was further challenged by language and cultural differences:

“They don’t understand the culture, they don’t understand in some cases their religious beliefs yes. Some people go in hospital and the doctor is telling you, you have got 2 days to live, you know what I mean?...... I have seen where people from Africa, from our continent go to the GP’s sometimes there is difficulty with language you know that first contacts with their reception is I have never seen it really good, so one of the things to maybe improve that, is provide information in different languages”. [MG, R4]

A minority of participants shared positive experiences of healthcare encounters, however these also stressed the importance of sincerity and building trust:

“My mum’s GP, the husband worked in the country, so she has also been in my country - so the thing is she has been in that environment so she is so helpful when it comes to African....there should be sincerity” [CG, R5]

Confidence in seeking an HIV test was also inhibited by the lack of perceived support for testing from key sources within local communities. Given the stigma that still surrounds HIV, it was suggested that community leaders need to act as role models, taking to action to show that HIV testing is an acceptable activity. Groups suggested that community acceptance could be supported through community-based outreach testing activities or simply through promotion of testing by community leaders:

“What you can do is call an event, and at this event we can say OK many people will come; when they come those people who are leaders they would start themselves. They will say “look this is a necessity and we have to check ourselves and this is private and there is no shame”….have some good speakers and then others will feel “OK, now I will do it”……everyone would follow.” [MM, R1]

There was also much discussion on the need for communities and professionals to work together, and that building a context of trust (through co-location in, and co-involvement of, local communities) would, in turn, enhance trust in, and support for, HIV testing activities of health professionals:

“I mean it is a kind of combination. You have got to get them to the health professionals. I agree with you that the community leaders have got a role to play and to pass on the message but also somebody professional (a doctor or nurse) needs to be there so people trust going for the test, trust the confidential system…because people can be embarrassed to talk about HIV so you need to bring professionals back to the community.” [MG, R4]

### Cues to action

This category includes views on existing HIV-related health promotion campaigns and suggestions for strategies that could encourage individuals to seek HIV testing. All groups suggested that not enough was being done to promote HIV testing, and suggested that raising awareness of HIV and providing information could be an effective way of encouraging people to get tested. The main criticism of current public health campaigns was their lack of visibility, for example on the television (TV) or radio:

“I have never seen an HIV campaign in Nottingham - and I’ve been here for 10 years.” [CL, R6]

All groups suggested that TV, radio, leaflets, emails and posters should be used to raise awareness about HIV testing and to enhance up to date knowledge about HIV. Some groups also suggested that Facebook and Twitter could be used, since people access these daily.

All groups discussed the relative merits of encouraging people to get tested through community outreach activities. A common view was that community outreach activities are more likely to be personally relevant, to be trusted and to be accessible. This was a strongly supported strategy and there were many different suggestions regarding how it might best be done (e.g. using community role models, having community events, using mobile testing facilities and eliciting support from religious institutions). In addition, some group members suggested that HIV testing during routine medical consultations and/or reminders to go for screening would help to overcome personal inhibitions – but this suggestion aroused great debate (related to perceptions of stereotyping identified above) and there was a lack of consensus on the issue:

“Well, I am rather grateful when they keep reminding me or calling me for anything…..I am very dependent on them, I was rather happy. Why not for HIV? [Referring to cervical cancer screening]…..when I received the first letter I didn’t care, but when they kept sending me letters I think for 4 or 5 times, I took it serious and then went there to do my check-up..” [CG, R3]

### Views on the proposed mHealth intervention

The groups were asked to reflect on the proposed mHealth intervention and to give their suggestions on its structure and content. On the whole, the concept of a text messaging intervention was very positively received and was seen as a potentially useful way of delivering up to date knowledge and information:

“I think it’s an easy excellent idea because the information will be stored in your mobile phone which is much easier than going to the GP or the hospital to get a leaflet. I think it is very good to have something like this to remind people where to go, where to access help in case they need it. I think it is quite important that we do that because most people around here haven’t got any time”. [MW, R2]

Perceived benefits included the fact that participants felt that they would have control over the intervention (e.g. they could choose whether to read or to delete messages). Receiving text messages was acknowledged to be quick and easy and would not require any time consuming processes (unlike, for example, attending a community awareness raising event). The brevity of text messages was also seen as a potential benefit for individuals who may have not have the time, inclination or literacy skills to read lengthier information sources:

“It should be short because when it’s a long text, some people are lazy to read. Just keep it short. I want also to add that the message should focus only on the most important points and avoid too much detail. Also, you can add a link in the message for more information so that whoever wants to read more can click on that link”. [YP, R4].

There were no particular concerns expressed over confidentiality or privacy, but due to the stigma and lack of trust around HIV and health services, groups stressed that it would be important that messages should come from a trusted source:

“I think there might be a concern if I don’t know who the sender is. If I got a message from someone who I don’t know saying that I need to go to a certain place to do the test, I think I might feel uncomfortable and just ignore or even delete the message. I might worry about data protection. The message needs to be sent from a trusted sender”. [MG, R7]

In addition, to be meaningful, groups suggested that messages should be personalized and tailored (e.g. using an individual’s name, delivering the messages in different languages and/or including community specific information):

“They ignore [other health campaigns] because they feel that they are separated from others. So, if the message is focused on a particular ethnicity or particular society, it will catch attention more. To speak to us personally, it is important to be in our native language.” [CG, R3]

Likewise, to tackle fear and lack of awareness, groups suggested that messages needed to have an upbeat, positive and reassuring emphasis and must include relevant information:

“Straight to the point - reminding you like you need to go for you HIV test but it shouldn’t be a command, it should be kind of polite. Yes if you say [Name] or Dear [Name] or Hi [Name] we are so and so, reminding you or encouraging you to go to such and such facility for your HIV test, remember it is free and you can also get such incentives like and tell them what there is, support for everyone…” [MM R1]

Potential concerns included whether individuals would get bored, alarmed or suspicious at receiving messages only about HIV:

“Will it be boring? I mean they might be interested for a few times and then they might try to stop you or ban you from texting maybe because they get bored…. Well this is my point, it is really quite a sensitive thing for example if you are bombarding somebody with just HIV every week or daily messages…..They might get paranoid. It would be good to have other information as well. Yes - I think the message content need to be diversified if you are going to send more than one for each individual. Don’t send the same message every time”. [YP, R3]

This issue was linked to the related concern that African communities might feel unfairly targeted or stereotyped around HIV:

“I think it is fair enough to remind people about their… health problems but it should be done in a way that doesn’t scare people and people don’t feel targeted for example if it is just African communities receiving the messages and if you know your colleagues and neighbors they are not receiving, it is it not good so it should be done in an intelligent way”. [CL, R5]

A suggested solution was to include messages on a range of other health topics so as to avoid an exclusive HIV-related focus.

There was a lack of consensus over the potential intervention structure (e.g. timing and frequency of messages or the length of the intervention). As noted above, one concern was that individuals should not feel ‘pestered’ by receiving too many messages. Another, related, concern was whether individuals would take the time to read or engage with the messages due to the sheer volume of phone input that individuals received everyday. Again, ensuring that the sender was known and trusted was suggested as a way of encouraging engagement with the texts:

“I just think you know, with phones, you receive all sorts of things now, people get annoyed with people trying to fix your windows and stuff and calling you, it is getting these messages and then there will be another campaign about something else, not to do with HIV, it just needs to be because the marketing the way it is kind of… you know bombarding messages just like to get you people know it just gets fed up. I mean I agree with you for example if the African Institute you receive something from it, it would only be known organization, we know the leaders we know who is running it so you are already accepting.” [CL, R4]

A summary of community recommendations and suggestions for intervention development are presented in Table 3.

**Table 3:** Recommendations for Message Development

Message dimension	Recommendations from the focus group discussions
Content	Messages should:
	• come from a trusted source
	• be in local languages
	• be personalised
	• be informative, encouraging and reassuring
	• not scare people
	• emphasise the benefits of knowing your HIV status
	• emphasise that testing is free for everyone
	• give information on where to get tested
	• not only focus on HIV, as this might get repetitive and might become boring
	• include information on other health issues
Structure	• No consensus on how often the messages should come, what time of day they should be received, or how long the intervention should last
	• Messages should not be too frequent, should not ‘bombard’ participants

### Intervention development

The section below shows how these key themes and suggestions were incorporated into the final intervention.

### Message structure

In terms of message frequency and duration of the intervention, no clear consensus emerged from the FGs. Therefore, the intervention was guided by existing evidence [9, 36].

The FGs clearly demonstrated discomfort of African communities about being singled out for HIV interventions. Hence, the intervention was given a generic name ‘Health4U’, and it was decided that one text per week would be on HIV and one text per week would be on a general health issue. In line with the evidence base [29], this strategy was also designed to address the concern expressed in FGs about possible boredom associated with too much repetition in messages.

The FGs highlighted the need for messages to come from a trusted source. Similar recommendations have been made in the literature [30]. The message sender was designated as ‘Health4U’, therefore each message was clearly identifiable as coming from the research project. In addition, the initial and final messages reminded participants that they were part of an AISD project (whereby AISD was considered the ‘trusted source’), however the character limits of SMS text meant that the reference to AISD could not be fitted into every message (see Table 4).

**Table 4:** HIV and general health messages and associated health belief model construct

Week	Message number	Message	Purpose	HBM constructs
1	1	Hi xxxxxxxxxx! Welcome to Health4U! You’ll get text messages for 12 weeks. Thanks! African Institute H4U	Welcome	
1	2	HIV is a virus which attacks the body. You can have it but not have symptoms. Without treatment you may develop AIDS. Visit www.nhs.uk/conditions/HIV/	HIV: Information on virus	PSus
1	3	Stress is normal; why not try Nelson Mandela’s advice for dealing with life’s ups and downs? “Tread softly, breathe peacefully, laugh hysterically”	General Health: Stress	
2	4	“Let food be your medicine and medicine be your food”. Why not try nuts, beans & whole grains as healthy snacks?	General Health: Diet	
2	5	Did you know that 3.8 % of Africans living in Nottingham have HIV? Knowledge is power – respect your body, protect your community and get tested.	HIV: Prevalence	PSev, SE, CTA
2	5 (2^nd^ option)	HIV can affect anyone. Latest statistics show HIV is rising in Muslim communities. Protect your community and get an HIV test.	HIV: Prevalence	PSus, CTA
3	6	A word of wisdom: “Wellness is a connection of paths: knowledge and action.” If you know you have a problem, don’t delay in seeking help.	General Health: Seek help	
3	7	HIV testing & treatment is free, confidential & anonymous for everyone! Visit http://nottingham.ac.uk/health4u for convenient places and times to get tested	HIV: Info on where to get tested	PBen, CTA
4	8	If you have HIV, the earlier you know, the sooner you can get treated. Get tested. It's free whatever your immigration status is.	HIV: Immigration	PBen, PBar, CTA
4	9	A person “who moves with each day is better than another who waits for luck”. Keep moving to keep fit! Anything helps: walking, dancing, even cleaning!	General Health: Exercise	
5	10	A word of wisdom: “Be the change you want to see in the world”. Encourage the ones you love to stay healthy.	General Health: Stay healthy	
5	11	Getting proper treatment soon after contracting HIV leads to a long and healthy life. Over half of Africans get diagnosed late – be on time & get a test!	HIV: Benefits of testing	PBen, PSev, CTA
6	12	“To keep the body in good health is a duty… otherwise we shall not be able to keep our mind strong and clear”. Eat well & exercise to keep calm and focused.	General Health: Stress	
6	13	Did you know you can go online to order an HIV test that will be sent to your home? It’s free and private! Visit http://www.tht.org.uk/sexual-health	HIV: Postal kit	PBar, CTA
7	14	Lots of support is here for you if you have HIV. The NHS has a confidential HIV service & http://www.tht.org.uk has info on support groups in Nottingham.	HIV: Support	SE, PB
7	15	Proverb: “One who eats alone cannot discuss the taste of the food with others”. A diet low in sugar, salt and fats will keep you and your loved ones healthy.	General Health: Diet	
8	16	If u or ur partner had unsafe sex in the past, ur at risk of carrying HIV. Test early to protect u & people u care for. Visit www.nottingham.ac.uk/health4u	HIV: Protecting others, test info	PSus, CTA
8	17	African Proverb: “He who conceals his disease cannot expect to be cured”. If you are not feeling well, don’t hesitate to see a doctor.	General Health: Seek help	
9	18	“It is health that is real wealth and not pieces of gold and silver”. Be active! Try to do at least 2½ hours of moderate physical activity each week.	General Health: Exercise	
9	19	Feeling good? Make sure you stay healthy & well and get an HIV test. Not everyone with HIV gets symptoms but early treatment can keep you feeling fit.	HIV: Feeling good	SE, PSus, PSev, CTA
10	20	African proverb: “If you close your eyes to facts, you will learn through accidents”. Find out how to stay healthy & feeling well. Visit www.nhs.uk/change4life	General Health: Stay healthy	
10	21	Look after yourself & your partner: Get tested together for HIV! People with HIV can still have great relationships. Visit http://StartsWithMe.org.uk	HIV: Protecting your partner, test info	SE, CTA, PBen
11	22	Many people in Nottingham have HIV and don't know it. Even if you feel well, it’s good to get tested at least once per year.	HIV: Invisible diagnosis	PSus, CTA
11	23	Proverb: “A friend is someone you share the path with”. Life can be stressful. Don’t be afraid to ask for help.	General Health: Stress/seek help	
12	24	“Health is the greatest possession. Contentment is the greatest treasure. Confidence is the greatest friend.” It’s important to look after your health.	General Health: Stay healthy	
12	25	Xxxxxxxxxx, thank you for taking part in Health4U! Health messages from us will now stop. Two more text messages will follow to ask your opinion on the project.	Thank you	
12	26	Have Health4U text messages inspired you to take action on health? Please reply YES if you have had an HIV test in the last 12 weeks & NO if not.	Did you get an HIV test?	
12	27	Was Health4U USEFUL for you? Please HELP our research by completing a short questionnaire. Visit www.nottingham.ac.uk/health4u. Thanks! African Institute H4U	Link to post-intervention survey	
12	26	Have Health4U text messages inspired you to take action on health? Please reply YES if you have had an HIV test in the last 12 weeks & NO if not.	Did you get an HIV test?	
12	27	Was Health4U USEFUL for you? Please HELP our research by completing a short questionnaire. Visit www.nottingham.ac.uk/health4u. Thanks! African Institute H4U	Link to post-intervention survey	

*PSus* perceived susceptibility

*PSev* perceived severity

*PBen* perceived benefits

*PBar* perceived barriers

*CTA* cue to action

*SE* self efficacy

Members of the FGs suggested that individuals would be most receptive to personalized messages in their own language. This is consistent with previous findings using text-messages to address HIV treatment adherence [18]. Messages were tailored to the 3 main spoken languages (English, French and Arabic) and religions (both asked of participants upon enrollment in the intervention). The “welcome” text message and the “end of the program” text message were personalized with the name indicated by participants.

The FGs showed no clear consensus regarding the best timing for the messages. Hence, the messages were delivered over the course of a week and delivered at different hours and times of the day in order to avoid habituation to message type (e.g. “it’s HIV message day/time”) and to facilitate action (e.g. messages related to diet habits were sent right before lunch).

### Message content

The HIV-related messages were constructed within the framework of the HBM (Table 4). The key themes within each of the HBM categories were reviewed and explicitly addressed within the messages. As in previous studies, most messages incorporated more than one HBM construct [43].

The non-HIV related texts were deliberately designed to be upbeat and motivational, drawing upon African proverbs where relevant in order to maintain interest (Table 4). These were focused on topical health themes including physical activity, nutrition, stress management, depression and the importance of regular check-ups with a GP.

The pre-testing process generated suggestions on how to make the meaning of messages clearer. For example, it showed that statistics needed to be used in messages with caution, since a number of participants misinterpreted them. After pre-testing, the revised messages were reviewed again by the entire project team and then agreed upon.

The final SMS intervention comprised 2 messages per week for a 12-week period. One SMS per week was related to HIV and one was related to a general health issue. The messages were tailored on religion, language and gender, and were personalized. The final 2 texts prompted participants to provide research outcome data by clicking on a link provided through the SMS and completing a questionnaire.

## Discussion

The FGs represent a wide and heterogeneous sample of Nottingham’s African communities. While a range of views were expressed, the findings strongly suggest a need to provide up to date, visible information about HIV and HIV testing in a culturally appropriate, locally relevant and supportive manner. These findings corroborate evidence from a 2014 national survey among African communities in the UK, which showed that even after considerable national investment into an HIV prevention campaign targeted to African communities (‘*It Starts with Me*’), 66.1 % of the national sample (*n* = 1,011) had not seen any materials or messages, and a further 13.6 % had seen some, but not read them [7].

In keeping with the wider literature in this area, the FG findings showed an ambivalent perception of healthcare in the UK that was acting as a barrier to health seeking around HIV [78]. Trust was identified as a key factor in facilitating any kind of response to messages around HIV [79]. A prominent theme related to a lack of trust in healthcare professionals but also a lack of trust in being able to discuss HIV and receive support within one’s own community. The findings suggest that community outreach and community involvement is essential in order to create a more enabling environment for discussion and action in relation to HIV, but also in order to demonstrate cooperative working relationships with health professionals, and thereby enhance community trust in the ‘system’. These results on the role of community involvement are consistent with findings from previous studies [80]. The prevailing context of mistrust and stigma surrounding HIV lends strong support to the mHealth intervention concept, by which individuals receive HIV-related information privately and are in control of who to discuss the texts with, and whether or not to take action.

The use of the HBM within the study enabled factors that influence HIV testing to be illuminated and explicitly addressed within the intervention. This was achieved by working closely with a trusted community organization, by providing relevant information, by providing reassurance about the quality of services offered and by not exclusively focusing on HIV.

### Study limitations and challenges

The study design brought key benefits but also challenges to the project. While the CBPR approach ensured that highly sensitive issues were openly discussed within the FGs, recruitment to the FGs may have been biased towards the Community Researchers’ existing social networks. This may have resulted in more informed or opinionated individuals taking part. In addition, the FGs had more female than male participants. The reasons for this are unclear, however the community researcher team suggested that this might have been due to men being more concerned about HIV stigma.

The decision to adopt a broad based approach to recruitment rather than sampling on the basis of previous HIV testing experience may have prevented more nuanced insights around testing from emerging. Nonetheless, despite these possible limitations, the FG findings are consistent with existing research in this field [4, 81, 82].

The collaborative nature of the project was essential for meeting the expressed needs of the community, but was a time and resource intensive process requiring careful management, as each step needed to be debated and agreed by a large group of stakeholders. This is one of the main challenges faced by researchers when conducting CBPR [45, 83]; as such, additional time for such engagement needs to be factored into the design of feasibility and formative studies.

Working collaboratively with the African community ensured that the HIV testing messages were culturally appropriate and relevant. However, this process was made more complex by the requirement to limit each SMS to 160 characters, in 3 languages and personalizing them. The character limit placed constraints upon the extent to which messaging could be creative and drafted in ways that drew upon common culturally-specific proverbs or slogans. The community’s strong recommendation to have Arabic and French versions of the messages generated additional challenges in ensuring that the translations (English, French and Arabic) were as consistent as possible, while considering the character limits and variations in acceptable grammar and syntax of the 3 languages [74].

Finally, despite widespread use of the HBM to inform the design of interventions to increase health screening uptake, the model has known limitations, stemming from its low predictive capability [84]. Future studies could be informed by recent theoretical approaches using validated taxonomies of behaviour change techniques (BCTs) to inform intervention design [85, 86].

## Conclusions

This paper has described a multi-stage, theory-based intervention research and development process. The potential influence of this intervention on promoting uptake of HIV testing amongst African communities has been evaluated in a feasibility study and the results reported elsewhere [51]. Definitive study outcomes need to be evaluated within the context of a randomized control trial. If shown to be effective, future research would need to be directed towards the application of the processes and methodologies described in this paper in other communities.

Finally, all those involved in the project reported personal growth and capacity development as a result of their engagement. Several community researchers reported that they had obtained new jobs or had embarked on new training programs, attributed in part to the skills and confidence they had gained from engagement in the research. Concurrently, the university team acquired a greater appreciation of, and respect for, community issues. This project successfully built a partnership that has been sustained and has led to the development of further collaborative research endeavors.

## Abbreviations

AISD, African Institute for Social Development; ART, Antiretroviral Therapy; BCT, Behavior Change Technique; CBO, Community Based Organization; CBPR, Community Based Participatory Research; CG, Christian Group; CL, Community Leaders; FG, Focus Group; GP, General Practitioner; HBM, Health Belief Model; HIV, Human Immunodeficiency Syndrome; HPA, Health Protection Agency; MG, Mixed Group; MM, Muslim Men; MW, Muslim Women; NHS, National Health Service; SMS, Short Message Service; TB, Tuberculosis; TV, Television; UK, United Kingdom; UNAIDS, United Nations Programme on HIV/AIDS; USA, United States of America; WHO, World Health Organization; YP, Young Persons

## Additional file

Additional file 1: Focus Group Topic Guide and Message Pre-Testing Elicitation Interview Questions. (DOCX 15 kb)
