# Erratum to: Developing a mHealth intervention to promote uptake of HIV testing among African communities in the UK: a qualitative study

**DOI:** 10.1186/s12889-016-3580-1

**Published:** 2016-09-08

**Authors:** C. Evans, K. Turner, L. S. Suggs, A. Occa, A. Juma, H. Blake

**Affiliations:** 1 School of Health Sciences, University of Nottingham, A Floor, South Block Link, Queen’s Medical Centre, Nottingham, NG7 2HA UK; 2 BeCHANGE Research Group, Institute for Public Communication, Faculty of Communication Sciences, Università della Svizzera italiana, Lugano, Switzerland; 3 School of Communication, University of Miami, Miami, USA; 4 African Institute for Social Development, Nottingham, UK

## Erratum

Upon publication, the following error was introduced to the title of this article [1]: The ‘UK’ was incorrectly changed to ‘conditions’.

The correct title should read: 'Developing a mHealth intervention to promote uptake of HIV testing among African communities in the UK: a qualitative study'.

The correct title has been included in this erratum and has also been updated in the original article.
