# Supplementary material for: Developing a mHealth intervention to promote uptake of HIV testing among African communities in the conditions: a qualitative study
Source: BMC Public Health. 2016 Jul 28;16:656. doi: 10.1186/s12889-016-3278-4 (PMC4964066; doi:10.1186/s12889-016-3278-4)
Supplement: Additional file 1: — Focus Group Topic Guide and Message Pre-Testing Elicitation Interview Questions. (DOCX 15 kb) [file 12889_2016_3278_MOESM1_ESM.docx]

**Supplementary Files**

**Focus Group Topic Guide**

VIEWS ABOUT HIV AND HIV TESTING

- What are your views on having tests to screen for different health conditions?
- In your opinion, what are the factors that prevent or discourage people in African communities from going to have health tests and screening?
- What things would encourage people in African communities to go for more health tests and check-ups?
- In your opinion, is HIV still a health problem for African communities in the UK?
- What can be done to encourage more people to go for HIV testing?
  - What are the things that stop people from going for an HIV test?
  - What would encourage people to have an HIV test?
- What are your views on existing HIV testing health promotion campaigns?
  - How could these be improved?
- What are your views on existing HIV testing services in Nottingham?
  - How could these be improved?

VIEWS ABOUT MOBILE PHONES AS A HEALTH INTERVENTION

- Tell us what you think about getting health promotion advice and messages through your mobile phone…...
- Would you have any concerns about getting health messages through your mobile phone?
  - And how could these concerns be overcome?
- What do you think are the most important messages to emphasise about HIV?
- What kind of messages would be acceptable to you?
  - How often?
  - At what time of day would you like to get messages like this?
  - What language should the messages be in?
  - Do you think men and women would need different messages?
- What would work as an appropriate incentive for people to participate in this research?

**Message Pre-Testing Elicitation Interview Questions**

- What is this message saying?
- What do you think this message is intended to mean?
- What does it tell you to do?
- Who do you think it is intended for? Do you think this message is relevant to you and others of a similar age, gender and cultural background?
- How do you feel about this message?
- What is the strongest message?
- What is the weakest message?
- Which one did you find most novel (original, unique, surprising)?
- Are there any messages that you had already heard about before?
